# The mechanism of directed Ni(ii)-catalyzed C–H iodination with molecular iodine†
†Electronic supplementary information (ESI) available: Additional geometrical information and free energy surfaces for the examined reaction pathways and computed energies in hartree for all reported structures. Cartesian coordinates for all reported structures are provided in xyz format (Struct.xyz). See DOI: 10.1039/c7sc04604a


**DOI:** 10.1039/c7sc04604a

**Published:** 2017-11-28

**Authors:** Brandon E. Haines, Jin-Quan Yu, Djamaladdin G. Musaev

**Affiliations:** a Cherry L. Emerson Centre for Scientific Computation , Emory University , 1515 Dickey Drive , Atlanta , Georgia 30322 , USA . Email: dmusaev@emory.edu; b Department of Chemistry , The Scripps Research Institute , 10550 North Torrey Pines Road , La Jolla , California 92037 , USA

## Abstract

The density functional theory method is used to elucidate the elementary steps of Ni(ii)-catalyzed C(sp^2^)–H iodination with I_2_ and substrates bearing *N*,*N*′-bidentate directing centers, amide-oxazoline (AO) and 8-aminoquinoline (AQ). The relative stability of the lowest energy high- and low-spin electronic states of the catalyst and intermediates is found to be an important factor for all of the steps in the reaction. As a result, two-state reactivity for these systems is reported, where the reaction is initiated on the triplet surface and generates a high energy singlet nickelacycle. It is shown that the addition of Na_2_CO_3_ base to the reaction mixture facilitates C–H activation. The presence of I_2_ in the reaction provides the much needed driving force for the C–H activation and nickelacycle formation and ultimately reacts to form a new C–I bond through either a redox neutral electrophilic cleavage (EC) pathway or a one-electron reductive cleavage (REC) pathway. The previously proposed Ni(ii)/Ni(iv) and homolytic cleavage pathways are found to be higher in energy. The nature of the substrate is found to have a large impact on the relative stability of the lowest electronic states and on the stability of the nickelacycle resulting from C–H activation.

## Abstract 2

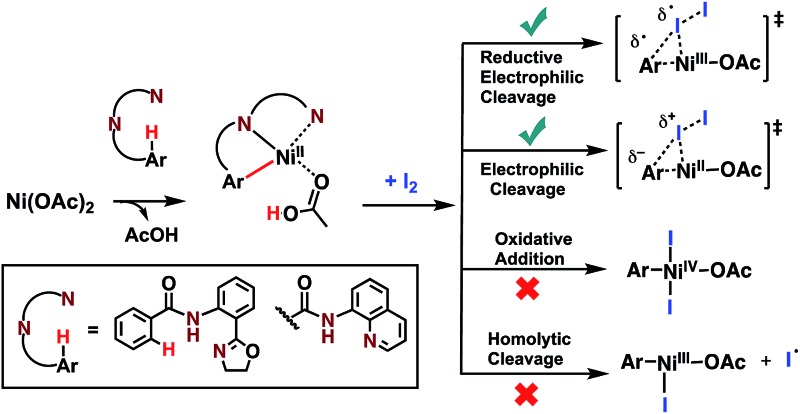
This computational study reveals electrophilic cleavage pathways for substrates with *N*,*N*-bidentate directing centers in Ni(ii)-catalyzed C–H iodination with molecular iodine.

## Introduction

Catalytic C–H functionalization—defined as the catalytic transformation of C–H bonds into C–B, C–C, C–N, C–O, C–S and C–halogen bonds—has inherent advantages for the development of environmentally friendly and sustainable synthetic routes to complex organic targets.^1^–^7^ Currently, many of the developments in this field rely on the use of expensive and rare noble metal catalysts, such as Au,^8^–^10^ Pt,^11^,^12^ Pd,^13^,^14^ Rh,^5^,^15^–^18^ and Ir.^6^,^18^,^19^ Therefore, the development of cost-effective earth-abundant transition metal catalysts (such as Fe, Co, Ni and Cu) is an attractive strategy to further capitalize on the sustainable potential of catalytic C–H functionalization.^20^–^22^ However, first-row transition metals, compared with their heavier analogues, suffer from (a) more complex reactivity (*i.e.* more accessible oxidation states and intermediates) due to their tendency to be involved in single-electron redox processes along with two-electron redox processes^23^ and (b) a lack of a driving force for insertion into C–H bonds because the resulting M–C and M–H bonds are weak.^24^ Thus, innovative approaches are necessary to design earth-abundant transition metal catalysts for C–H functionalization.

Existing strategies in this field of scientific research employ photoredox^25^–^28^ or chelation assisted (*i.e.* directing group, DG, assisted)^29^–^47^ approaches. These studies have unambiguously demonstrated the effectiveness of substrates with two chelating centers, such as 8-aminoquinoline (AQ), picolinamide (PA) and others, to direct the C–H activation event.^48^,^49^ It is believed that the bidentate coordination of the substrate to the metal center provides stability to the pre-reaction complex and brings the activated C–H bond in close proximity to the transition metal center.^50^,^51^ Furthermore, these studies have identified the utmost importance of controlling the multitude of oxidation states of the transition metal centers in the course of the reaction, which may proceed *via* numerous pathways such as: (a) a two-electron redox pathway (*i.e.* oxidative addition and reductive elimination), (b) a single-electron oxidation/reduction pathway (for example, *via* reactive organic radical intermediate formation) and (c) redox neutral pathways.^23^

Several recently reported computational studies have supported the above-mentioned complexity of Ni(ii)-catalyzed C–H functionalization reactions. Omer and Liu have shown that while the C(sp^2^)–H and C(sp^3^)–H bond cleavage of substrates with an 8-aminoquinoline (AQ) group by Ni(ii)-catalyst occurs *via* the concerted metalation–deprotonation (CMD) mechanism,^52^,^53^ the mechanism of the subsequent C–C and C–X bond formation steps depends on the nature of the substrate and the coordination environment of the metal. They may occur *via* either radical mechanisms (involving Ni(iii) complexes) when the coupling partners are substrates with steric hindrance and low X–Y/X bonding energies, such as dicumyl peroxide (O–O bond), heptafluoroisopropyl iodide (3° alkyl C–I bond) and diphenyl disulfide (S–S bond), or *via* an oxidative addition/reductive elimination mechanism involving a Ni(iv) intermediate when the coupling partners are phenyl iodide (aryl C–I bond) and *n*-butyl bromide (1° alkyl C–Br bond).^54^ Sunoj and coworkers also report that aryl iodides react through a Ni(ii)/Ni(iv) mechanism with C(sp^3^)–H AQ substrates, where the regioselectivity is determined by the reductive elimination step.^55^ Importantly, they demonstrate that the modeling of additives in the reaction can have a large impact on the computed pathways. Thus, the nature of the coupling partners (oxidants), transition metal centers and additives, as well as both the nature and number of chelating centers, are vital for C–H functionalization using first-row transition metal catalysts.

**Fig. 1:**
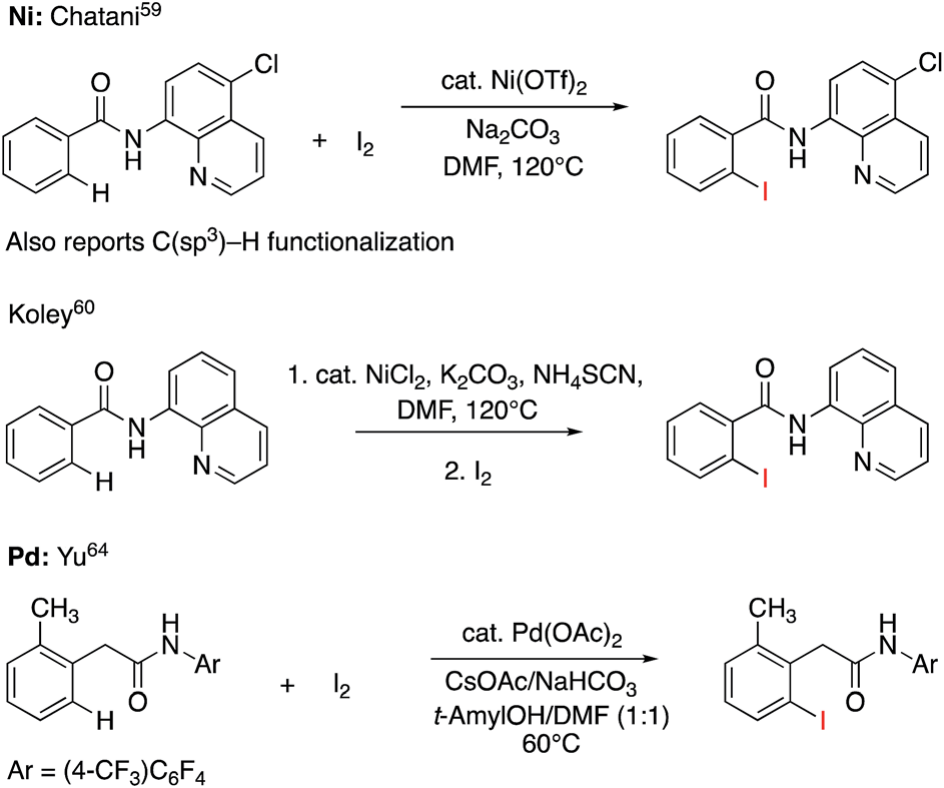
Recent representative developments in Ni(ii)- and analogous Pd(ii)-catalyzed C–H iodination reactions with I_2_ as the sole oxidant.

**Fig. 2:**
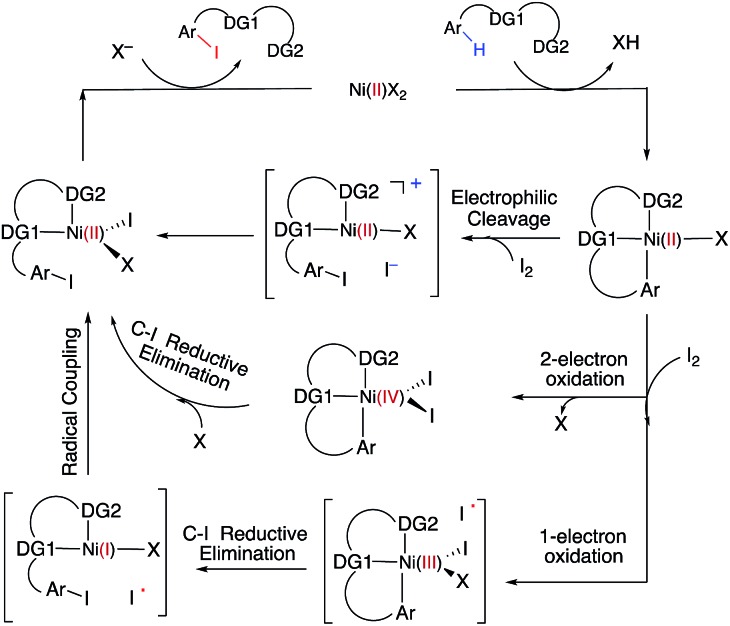
Proposed mechanisms for Ni(ii)-catalyzed C–H iodination reactions with I_2_.

C–H iodination with molecular I_2_ under mild experimental conditions is a highly desirable process because it utilizes inexpensive I_2_ as the sole oxidant and increases the accessibility of synthetically valuable aryl halide compounds. The design of the first-row transition metal catalysts for this reaction is expected to be even more challenging because of the amphiphilic or “chameleon” nature of the I_2_ molecule, which can act as either an electron-donor (L-type) or electron-acceptor (Z-type) ligand in transition metal complexes.^56^–^58^ As a major advancement in this field, the Ni(ii)-catalyzed C–H iodination of an AQ substrate with I_2_ with *N*,*N*′-directing groups was recently reported by both Chatani and coworkers^59^ and Koley and coworkers^60^ (Fig. 1). However, the mechanism of this reaction has not yet been studied in detail. Koley and coworkers proposed that either Ni(ii)/Ni(iv) or redox-neutral Ni(ii)/Ni(ii) mechanisms could be operative (Fig. 2). In contrast, Chatani and coworkers settled on a Ni(ii)/Ni(iii) redox cycle that was previously proposed by Sanford and coworkers^61^,^62^ for C–Br bond formation from the reaction of Br_2_ and Ni(ii)(phpy)(Br)(pic), where phpy = 2-phenylpyridine and pic = 2-picoline. A notable computational study by Hall and coworkers predicted a Ni(ii)/Ni(iii) spin-crossover mechanism for the C–Ni bromination reaction.^63^

To deal with the mechanistic complexity of Ni(ii)-catalyzed C–H iodination with I_2_, the knowledge acquired from the analogous Pd(ii)-catalyzed reaction^64^ could be useful, despite known differences in the electronic structure and reactivity of the Ni(ii) and Pd(ii) species.^65^,^66^ In their seminal work, Yu and coworkers used a commercially available monodentate acidic amide DG for the Pd(ii)-catalyzed reaction,^64^ as opposed to the *N*,*N*′-bidentate directing groups used for the Ni-catalyzed reaction (Fig. 1). Our following extensive computational study^56^ of this reaction revealed that C–I bond formation proceeds *via* a redox-neutral electrophilic cleavage (EC) mechanism initiated by the coordination of I_2_ as a Z-type ligand^57^,^67^,^68^ to the axial position of the square-planar d^8^ Ar–Pd(ii) C–H activation intermediate.^56^ Its two-electron Pd(ii)/Pd(iv) oxidation mechanism, including (a) I–I oxidative addition to the Ar–Pd(ii) intermediate and (b) C–I reductive elimination from the resulting Pd(iv) intermediate, is less favorable.^69^–^71^ In addition, recently we have shown that the presence of a mono-N-protected amino acid ligand (MPAA) changes the mechanism by enabling the oxidation of the Pd(ii) center by I_2_ prior to C–H activation.^72^

**Fig. 3:**
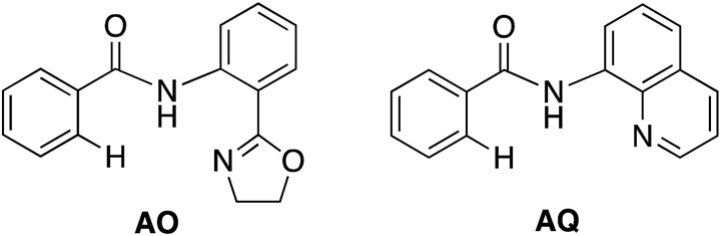
The substrates, AO and AQ, with *N*,*N*′-bidentate directing groups investigated in this paper.

With this uncertainty surrounding the mechanism, its importance for first-row transition metal catalyst design and the available knowledge in the literature, here we use computational methods to explore the possible mechanisms and governing factors of Ni(ii)-catalyzed C–H bond iodination by molecular I_2_ for substrates with *N*,*N*′-bidentate chelating groups, amide-oxazoline (AO) and AQ substrates (see Fig. 3). One should note that the AO ligand, developed in the Yu group, was previously used successfully for Cu-catalyzed C–H functionalization.^73^–^77^ Here, we chose a common Ni(ii) source, Ni(OAc)_2_, as a model catalyst because we aim to answer general questions about the reactivity of Ni(ii) catalysts in C–H activation and iodination with I_2_. It is recognized that the identity of the pre-catalyst and the mechanism for entering the catalytic cycle are critical aspects of a successful reaction, but this is not the major focus of this study. Therefore, we do not strive for direct correlation with all of the successful experimental reaction conditions but instead focus on the general conclusions for how the catalyst achieves the critical C–H activation and iodination steps.

In general, the results of this study align with those reported by Liu^54^ and Sunoj^55^ but the reactivity of I_2_ and the redox neutral EC pathway were not previously studied computationally. Furthermore, the previously reported studies^50^,^51^,^54^,^55^ did not fully elaborate on the impact of the lowest-lying electronic states of the catalyst and intermediates in the mechanism. Therefore, here, for the first time in the literature, we carefully analyze the impact of the lowest-lying singlet and triplet electronic states in Ni-catalyzed C–H functionalization. It is expected that this fundamental understanding of Ni-catalyzed C–H iodination reactions and the comparison of the acquired knowledge with that from the previously studied Pd-catalyzed reaction will enhance our ability to design cost-effective and environmentally friendly Ni-catalyzed C–H functionalization reactions and open new avenues for the design of first-row transition metal catalysts for C–H halogenation.

## Results and discussion

### Ni(ii)-catalyzed C–H iodination of the amide oxazoline (AO) substrate with I_2_

#### Mechanism of C–H activation

**Fig. 4:**
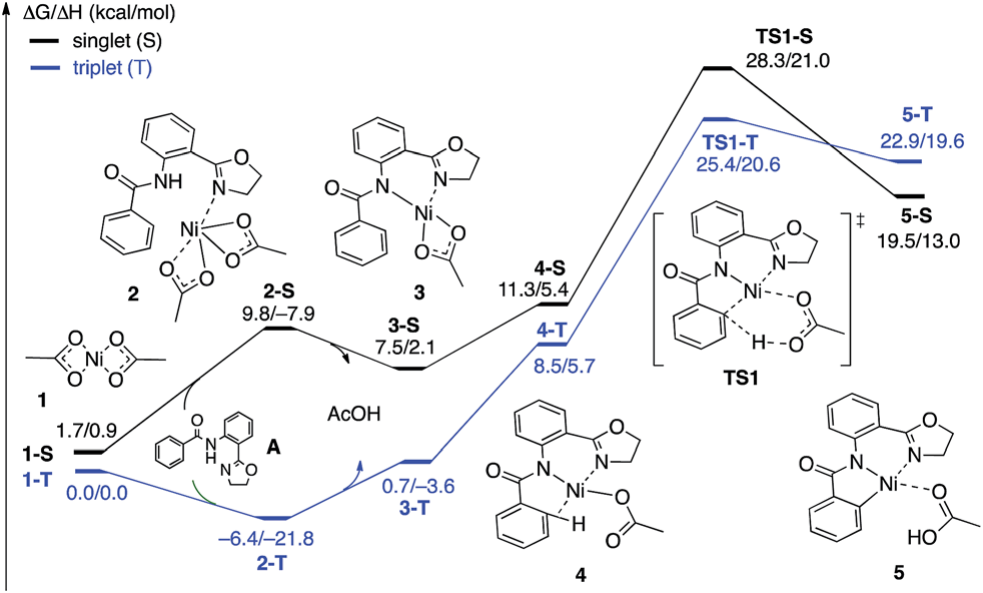
The singlet (black) and triplet (blue) free energy surfaces for the C–H bond activation of the AO substrate starting from the Ni(OAc)_2_ catalyst. Energies are reported as Δ*G*/Δ*H* in kcal mol^–1^.

Our extensive calculations (see Fig. 4 and the ESI†) show that the reaction of Ni(OAc)_2_ with the AO substrate is a multi-step process that proceeds *via* a triplet ground electronic state for the reactants, intermediates and two concerted metalation–deprotonation (CMD) transition states (for the deprotonation of N–H and C–H bonds, sequentially) but leads to the singlet state nickelacycle (**5-S**). Thus, it is most likely that the singlet and triplet surfaces of the reaction cross and both of the electronic states of the system contribute to the reactivity.

The first CMD process (*i.e.* the deprotonation of the amide, which was not calculated) and the subsequent dissociation of acetic acid completes the bidentate coordination of the substrate to the Ni center with its two chelating N-groups. In the resulting (AO-*k*^2^-*N*,*N*′,*CH*)Ni(ii)(OAc) complex, **3**, the *ortho*-C–H bond of the phenyl group in the substrate is closely positioned to the Ni center. Subsequently, cleavage of this *ortho*-C–H bond by the second acetate ligand through the CMD transition state, **TS1**, leads to the formation of the nickelacycle (AO-*k*^3^-*N*,*N*′,*C*)Ni(ii)(AcOH), **5**, with two Ni–N bonds and one Ni–C bond. C–H bond deprotonation at the transition state **TS1** is found to be the rate-limiting step of the process and occurs with a 31.8 kcal mol^–1^ free energy barrier (on the triplet state PES). Since the ground electronic state of **TS1** is a triplet state, but that of nickelacycle **5** is a singlet state, it is most likely that the singlet and triplet surfaces of the reaction cross after the triplet state C–H activation transition state. Thus, this process involves two lower-lying electronic states of the reactants, intermediates and transition states (*i.e.* shows two-state reactivity^78^). Thus, the formation of nickelacycle **5** is endergonic by 25.9 kcal mol^–1^ (Fig. 4).

The computed thermodynamic instability of the nickelacycle (AO-*k*^3^-*N*,*N*′,*C*)Ni(ii)(AcOH) relative to reactants Ni(OAc)_2_ (triplet) + AO is consistent with a previous computational study on the oxidative addition of C–H bonds to Ni(0) complexes.^79^ This is also consistent with the deuterium labeling experiments performed by Chatani and coworkers,^59^ the experiments of Koley and coworkers demonstrating that the nickelacycle formed by C–H activation cannot be isolated in the absence of I_2_,^60^ as well as the computational findings by Chen^50^,^51^ and Liu.^54^ Additional support for this conclusion comes from the fact that nickelacycles achieved *via* C–H activation are rare in the literature.^80^ This is in contrast to analogous Pd-catalyzed reactions, where the palladacycles resulting from C–H activation are thermodynamically stable and can often be isolated, characterized and used as pre-catalysts.^81^–^83^ Thus, based on the results given above, we once again highlight one of the foremost difficulties for Ni(ii)-catalyzed C–H functionalization as the lack of a thermodynamic driving force for C–H activation,^84^ which is a major reason for the failure of the isolation and characterization of nickelacycles from C–H activation processes. Of course, in the presence of an oxidant/electrophile, for example I_2_, the C–H formation barrier (*i.e.* the reverse barrier for C–H activation) is expected to compete with either I–I bond activation (if the reaction proceeds *via* an oxidative addition mechanism) and/or C–I bond formation (if the reaction proceeds *via* an electrophilic addition mechanism) and/or radical formation barriers. These processes are discussed in the next section.

**Fig. 5:**
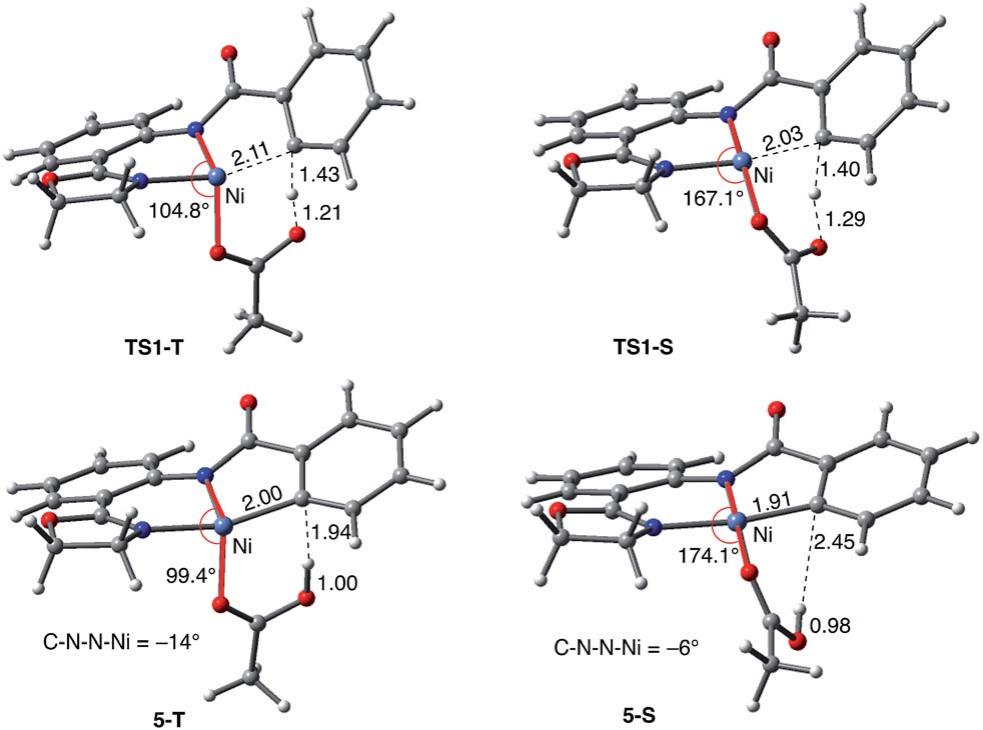
The structures of the C–H activation transition states (**TS1-T** and **TS1-S**) and products (**5-T** and **5-S**) on the triplet and singlet surfaces. Distances and angles are shown in Å and deg., respectively.

As shown in Fig. 5, the electronic state of the system has a significant impact not only on the energetics but also on the geometry of the C–H activation transition states and products. The most striking difference is in the angle of the acetate base relative to the substrate coordination plane: in the triplet structures, **TS1-T** and **5-T**, the acetate is nearly perpendicular to the substrate coordination plane (N–Ni-OAc = 104.8° and 99.4°, respectively), whereas in the singlet structures, **TS1-S** and **5-S**, the acetate is in the plane of the substrate (N–Ni-OAc = 167.4° and 174.1°, respectively). It is also noted that the AO substrate is capable of twisting slightly away from planarity (in **5-T**, C–N–N–Ni = –14°), which may allow for some stabilization of the transition state toward the tetrahedral geometry favored on the triplet surface.^63^

#### Role of sodium carbonate additive

Since available experiments^59^,^60^ have shown that the addition of Na_2_CO_3_ base into the reaction mixture improves both the reaction yield and reaction time of Ni(ii)-catalyzed C–H iodination in substrates with *N*,*N*′-bidentate directing groups, here, we also investigated the rate-limiting C–H activation step of this reaction in the presence of Na_2_CO_3_. In general, previous studies have indicated that the base additive may influence the C–H activation step through (a) ligand exchange reactions that lead to the *in situ* formation of a different catalyst,^85^,^86^ (b) scavenging protons or acetic acid to drive the C–H activation,^87^–^90^ and/or (c) the formation of a molecular cluster with other components (substrate, ligand, solvent, *etc.*) of the reaction that can promote the C–H activation step either *via* direct involvement in the CMD transition state or through non-covalent interactions with the substrate.^91^,^92^

**Fig. 6:**
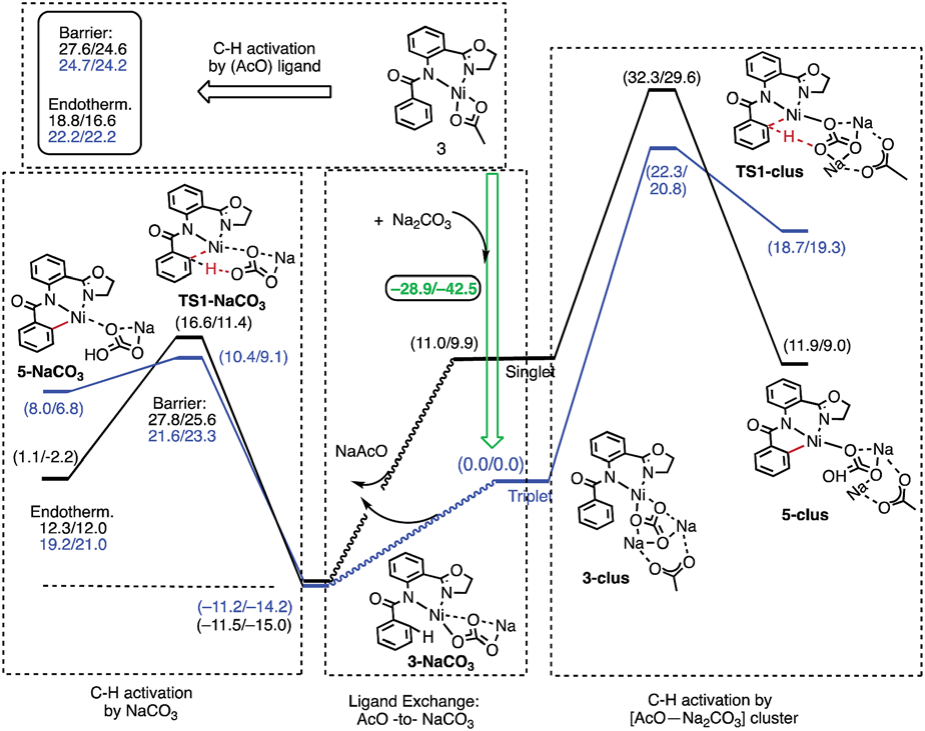
Schematic presentation of the elementary reactions involved in the addition of Na_2_CO_3_ to complex (AO-*k*^2^-*N*,*N*′,*CH*)Ni(ii)(OAc), **3**. All of the given energies are calculated relative to the triplet electronic states of the corresponding pre-reaction complexes. The energies are presented as Δ*G*/Δ*H* and are in kcal mol^–1^.

The results presented in Fig. 6 show that the addition of the Na_2_CO_3_ molecule to complex **3** leads to the formation of (AO-*k*^2^-*N*,*N*′,*CH*)Ni(ii)(OAc···Na_2_CO_3_), (**3-clus**) “(AcO···Na_2_CO_3_)-cluster-complex” (see ESI, Fig. S1,† for selected geometries); the calculated free energy of the reaction (AO-*k*^2^-*N*,*N*′,*CH*)Ni(ii)(OAc) + Na_2_CO_3_ → (AO-*k*^2^-*N*,*N*′,*CH*)Ni(ii)(OAc···Na_2_CO_3_) is –28.9 kcal mol^–1^. This complex has a triplet ground electronic state with 1.61|*e*| alpha-spin density on the Ni centers. Its open-shell singlet state (with an *S*^2^ value of 0.63) is 11.0 kcal mol^–1^ higher in free energy. From the triplet “cluster-complex” **3-clus**, the reaction may proceed either *via* C–H bond activation through the CMD triplet transition state **TS1-clus** by the (AcO···Na_2_CO_3_) ligand, or *via* ligand-exchange (*i.e.* NaOAc dissociation) where the subsequent C–H bond activation requires almost no barrier (we were not able to locate the associated transition state) and is exergonic by 11.5 kcal mol^–1^. Thus, one may confidently conclude that the addition of a Na_2_CO_3_ molecule to the reaction mixture will produce AcO-to-NaCO_3_ ligand exchange and will generate the new catalytically active species (AO-*k*^2^-*N*,*N*′,*CH*)Ni(ii)(NaCO_3_). In this newly generated active species, C–H bond activation requires a 21.6 kcal mol^–1^ free energy barrier and is endergonic by 12.3 kcal mol^–1^. A comparison of these energy parameters for active species (AO-*k*^2^-*N*,*N*′,*CH*)Ni(ii)(NaCO_3_) with those for (AO-*k*^2^-*N*,*N*′,*CH*)Ni(ii)(OAc) (calculated relative to **3-T**), a 24.7 kcal mol^–1^ free energy barrier and 18.8 kcal mol^–1^ endergonicity, clearly demonstrates the benefits of the presence of Na_2_CO_3_ in the reaction conditions. This is consistent with the findings of Liu and coworkers that a Ni(NaCO_3_)_2_·4DMF catalyst is the likely active catalyst in their systems.^54^ To summarize, the addition of Na_2_CO_3_ to the reaction mixture (a) generates the new catalytically active species (AO-*k*^2^-*N*,*N*′,*CH*)Ni(ii)(NaCO_3_) with a small or no energy barrier, (b) reduces the rate-limiting C–H activation barrier by 3.1 kcal mol^–1^ and (c) stabilizes the C–H activation product by 6.3 kcal mol^–1^.

#### Mechanism of iodination with I_2_

**Fig. 7:**
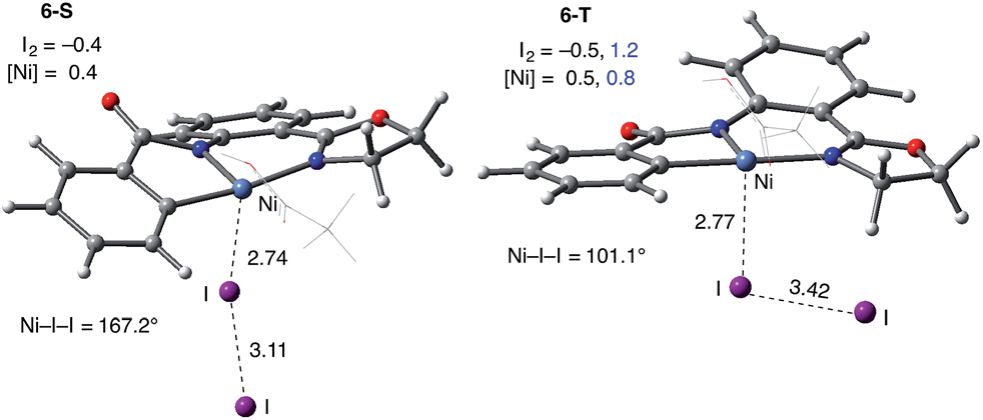
Structural features of the I_2_ coordination complexes (**6-S**) and (**6-T**). The distances are in Å, the angles are in degrees and the fragment Mulliken charge and spin density (in |*e*|) are shown in black and blue, respectively. For simplicity of presentation, the AcOH ligand is shown in the wireframe representation.

The next step of the reaction of the substrate AO with I_2_ is the addition of the oxidant to nickelacycle **5-S**. This process is found to be thermodynamically favorable, provides additional stabilization to the C–H activation product and, consequently, increases the barrier of the reverse reaction (*i.e.* C–H bond formation). A similar result was previously reported for I_2_ addition to a palladacycle in our study on the analogous Pd(ii)-catalyzed reaction.^56^ The coordination of I_2_ to the axial position of nickelacycle **5-S** to form **6-S** is exergonic by 9.4 kcal mol^–1^. However, it is still endergonic by 10.1 kcal mol^–1^ relative to the dissociation limit of **1-T** + AO + I_2_. The geometric signatures of the resulting complex **6-S**—the elongation of the I–I bond from 2.87 Å in free I_2_ to 3.11 Å in **6-S** and the linearity of the interaction between Ni and I_2_ (with ∠Ni–I–I = 167.2°)—imply the donation of a partial electron from the Ni d_*z*^2^_ orbital into the I_2_ σ* orbital^56^,^58^ (see Fig. 7).

Consistently, the triplet electronic state of the I_2_ coordination complex (**6-T**) becomes only 0.7 kcal mol^–1^ higher in free energy than its singlet state counterpart **6-S**. Analysis of unpaired spin densities (with 1.2|*e*| and 0.8|*e*| on the I_2_ and Ni fragments, respectively) allows us to characterize **6-T** as a Ni(iii)–I_2_ complex formed by one electron transfer from the Ni center to I_2_ (the calculated Mulliken charges of the I_2_ and Ni fragments are –0.5|*e*| and 0.5|*e*|, respectively).^63^ As a result of this full (rather than partial) Ni-to-I_2_ electron transfer, the elongation of the I–I bond (3.42 Å) becomes more pronounced than in **6-S** and the ∠Ni–I–I angle becomes bent (101.1°).

**Fig. 8:**
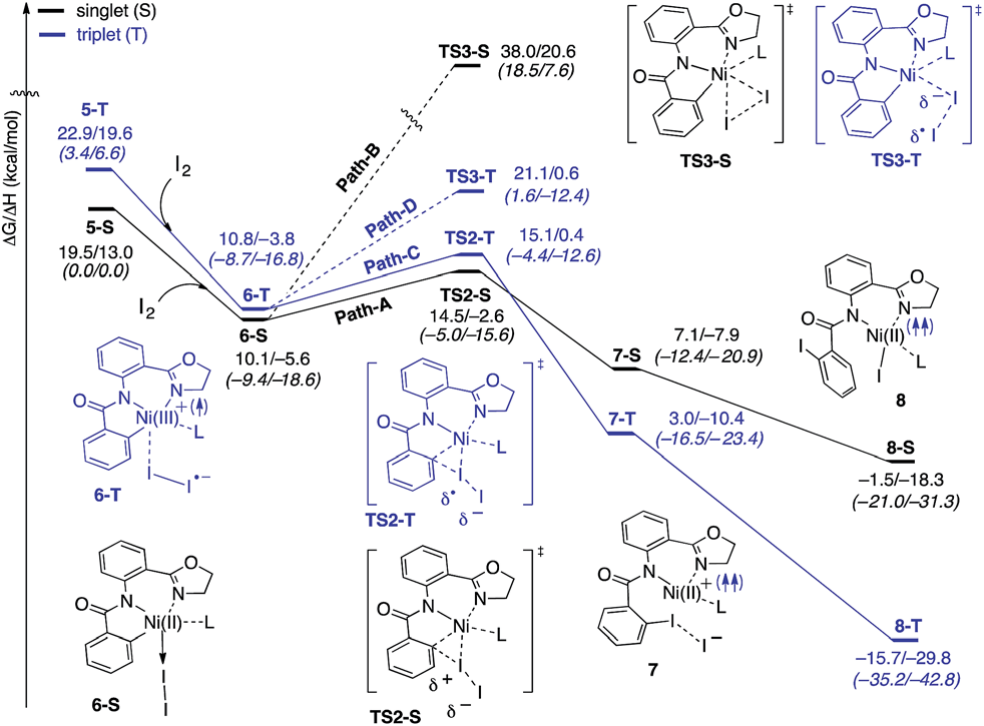
The singlet (black) and triplet (blue) free energy surfaces for the C–H bond iodination of the AO substrate. The energies are reported as Δ*G*/Δ*H* in kcal mol^–1^. The numbers given in the first and second lines are relative to the dissociation limits of Ni(AcO)_2_ (triplet) + AO + I_2_ and **5-S** + I_2_, respectively. Here, L stands for AcOH.

The accessibility of the triplet state complex **6-T** upon single electron transfer from Ni to I_2_ makes the reactivity of the nickelacycle with I_2_ more complex than that of its Pd analogue. Indeed, as illustrated in Fig. 8, one can expect two distinct iodination pathways for each of the singlet and triplet state complexes **6-S** and **6-T**. For the singlet **6-S** complex, the pathways are analogous to those studied for the Pd-catalyzed reaction: (A) a redox neutral Ni(ii)/Ni(ii) pathway proceeding through the concerted electrophilic cleavage (EC) of I_2_ and concomitant C–I bond formation (black solid line in Fig. 8) and (B) a Ni(ii)/Ni(iv) pathway proceeding through I–I oxidative addition (OA) followed by C–I reductive elimination (black dashed line in Fig. 8). For the triplet state **6-T** complex, these pathways are (C) a single electron reductive electrophilic cleavage (REC) Ni(iii)/Ni(ii) process in which C–I bond formation and the one electron reduction of the Ni(iii) center occur simultaneously (blue solid line in Fig. 8), and (D) a radical mechanism (RA) in which cleavage of the I–I bond forms a Ni(iii) complex and iodine atom (blue dashed line in Fig. 8). It is possible that these pathways can interconvert into each other by crossing between the singlet and triplet surfaces. Below, we discuss these processes in more detail (for the sake of simplicity, the free energies discussed in this section are calculated relative to the singlet state complex **6-S**).

#### Path-A: redox neutral Ni(ii)/Ni(ii) electrophilic cleavage (EC) mechanism

**Fig. 9:**
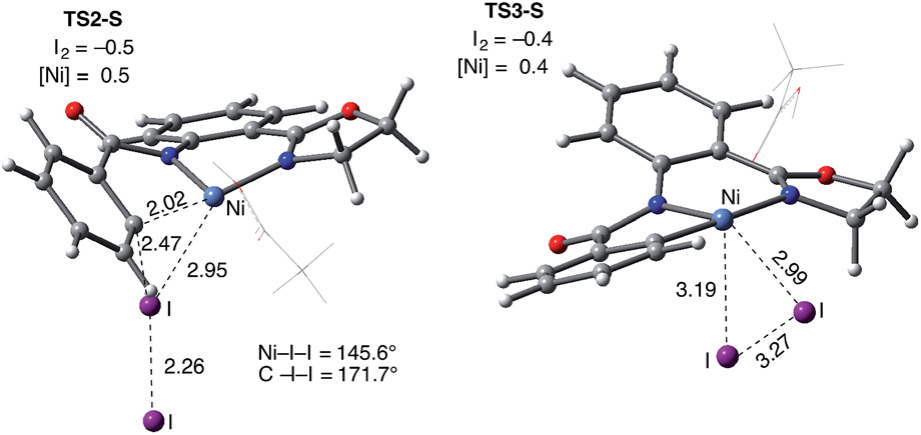
Structural features of the iodination transition states on the singlet surface, electrophilic cleavage (**TS2-S**) and oxidative addition (**TS3-S**). The distances are shown in Å, angles in degrees and fragment Mulliken charges in |*e*|. For simplicity of presentation, the AcOH ligand is shown in the wireframe representation.

This pathway of the reaction is initiated by the electrophilic attack of I_2_ on the Ni(ii)–C bond at the transition state (**TS2-S**). As shown in Fig. 9, at **TS2-S** the proximal iodonium engages in bonding with the Ni and C centers (with Ni–I = 2.95 Å and I–C = 2.47 Å) while the terminal iodide is displaced (with I–I = 3.26 Å). The free energy barrier associated with this transition state is only 4.4 kcal mol^–1^, which is much smaller than the overall 16.0 kcal mol^–1^ barrier required for the reverse C–H activation (*i.e.* C–H bond formation) (see Fig. 4 and 8). Thus, the addition of I_2_ to the reaction mixture of Ni(OAc)_2_ and the AO substrate makes C–H activation and, consequently, C–H iodination irreversible. IRC calculations initiated from the transition state (**TS2-S**) show that in the EC product (**7-S**) the C–I bond is formed and the expelled iodide forms an ion-pair with a Ni(ii)^+^ center. Formation of **7-S** is exergonic by 3.0 kcal mol^–1^ and the combination of the Ni(ii)^+^ and iodide ions to produce the Ni(ii)–I intermediate **8-S** is exergonic by 8.6 kcal mol^–1^.

#### Path-B: Ni(ii)/Ni(iv) 2-electron oxidation pathway

This pathway starts with the oxidative insertion of Ni(ii) into the I–I bond at the transition state **TS3-S**, where the breaking I–I bond is I–I = 3.27 Å, but the forming I_eq_–Ni and I_ax_–Ni bonds are 2.99 and 3.19 Å, respectively (see Fig. 9). The free energy barrier associated with this oxidative addition transition state is 27.9 kcal mol^–1^, which is 23.5 kcal mol^–1^ higher than that required for the electrophilic cleavage (EC) pathway (Path-A) (see Fig. 8). These conclusions for the EC and OA pathways are consistent with our previous study on Pd-catalyzed C–H iodination where the redox neutral Pd(ii)/Pd(ii) pathway is also shown to be more favorable than the Pd(ii)/Pd(iv) mechanism.^56^ Since the OA pathway cannot compete with the EC pathway, here we will not discuss this OA pathway in more detail, while we have included full computational data on the OA pathway in the ESI (see Fig. S2†). We also investigated the possibility of dissociation of L, AcOH in this case, from **6-S** to facilitate oxidative addition. We found this process to have a slightly lower overall barrier (**6-S** → **TS3-S-I2**, Δ*G*^‡^ = 25.2 kcal mol^–1^) than the reaction through **TS3-S** (see ESI for more details†). Regardless, this reaction pathway is much higher than the EC pathway.

#### Path-C: Ni(ii)/Ni(iii) single electron reductive electrophilic cleavage (REC)

**Fig. 10:**
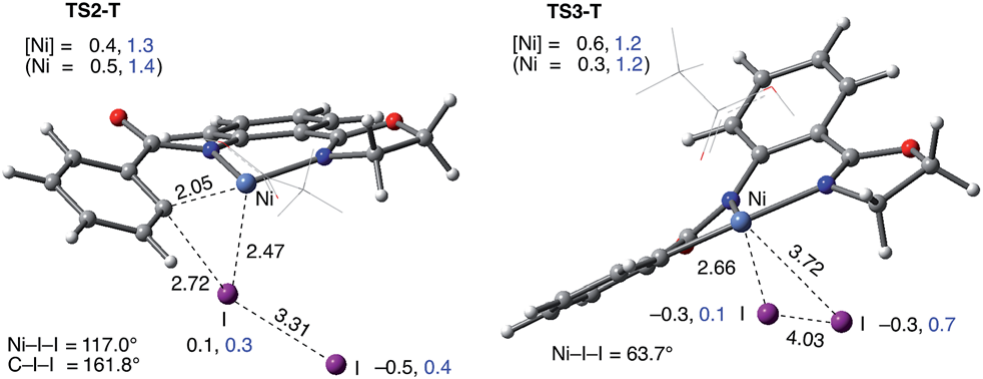
Structural features of the iodination transition states on the triplet surface, REC (**TS2-T**) and RA (**TS3-T**). The distances are shown in Å, angles in degrees and the fragment Mulliken charge and spin density (in |*e*|) are shown in black and blue, respectively. For simplicity of presentation, the AcOH ligand is shown in the wireframe representation.

The one-electron oxidation process of converting **6-S** to **6-T** (*i.e.* Ni(iii)^+^–I_2_^–^ complex) initiates this pathway. In the next step, the Ni(iii)^+^–C bond abstracts an iodine atom from I_2_^–^, which reduces the Ni(iii) center and releases iodide. Thus, this pathway couples the one-electron reduction of the metal with electrophilic cleavage (REC). Mulliken charge and spin density analysis of the I_2_ (–0.4|*e*| and 0.7|*e*|) and Ni (0.4|*e*| and 1.3|*e*|) fragments of the associated transition state (**TS2-T**) shows spin density transfer from I_2_^–^ to the Ni complex (Fig. 10). Of particular interest is that the distal I has a significant negative charge (–0.5|*e*|) while the proximal I does not (0.1|*e*|). Overall, the geometry of **TS2-T** is similar to **TS2-S** except that the Ni center and I_2_^–^ have a bent geometry (Ni–I–I = 117.0°) and the proximal iodine atom interacts more closely with the Ni center (I–Ni = 2.47 Å). Like in **TS2-S**, the terminal iodide is displaced (I–I = 3.31 Å) while the new I–C bond is forming (I–C = 2.72 Å). The free energy barrier (calculated relative to complex **6-S**) for the REC pathway is found to be 5.0 kcal mol^–1^, which is 0.6 kcal mol^–1^ higher than the EC pathway on the singlet surface. The product complex, **7-T**, is an ion-pair between Ni(ii)^+^ and iodide and is analogous of **7-S** except that the Ni(ii) is high-spin (there is a spin density of 1.62|*e*| on the Ni center). The formation of **7-T** is exergonic by 7.1 kcal mol^–1^ and the combination of the Ni(ii)^+^ and iodide ions to produce **8-T** is exergonic by 18.7 kcal mol^–1^.

With the expulsion of I^–^ during the reaction, we also investigated the role of I_3_^–^ complex formation in the presence of excess I_2_. We compute I_3_^–^ formation from I_2_ and I^–^ to be exergonic by 12.3 kcal mol^–1^. This suggests that I_3_^–^ complex formation could be playing the role of providing additional driving force for I^–^ generation. Indeed, coordination of an additional molecule of I_2_ to complexes **7-S** and **7-T** to form **7-S-I3** and **7-T-I3**, respectively are exergonic by 15.6 and 13.0 kcal mol^–1^, respectively. Thus, we propose that the EC and REC pathways can be facilitated by I_3_^–^ formation.

#### Path-D: Ni(ii)/Ni(iii) single electron radical pathway (homolytic cleavage)

This pathway is also initiated by the Ni(iii)^+^–I_2_^–^ intermediate, **6-T**. In the next step, the I–I bond of I_2_^–^ is cleaved through iodide abstraction by the cationic Ni(iii) center (*i.e.* charge recombination) to produce a Ni(iii)–I intermediate and iodine atom. Here we refer to this pathway as the radical pathway (RA), but it is analogous to the homolytic cleavage pathway described by Liu.^54^ In the associated transition state (**TS3-T**), Mulliken spin density analysis of the I_2_ fragment shows that the distal I has significant radical character (0.7|*e*|), while the proximal I has little (0.1|*e*|) (Fig. 10). In the TS, the bond between the proximal iodine atom and the Ni center is almost fully formed (I–Ni = 2.66 Å) and the distal iodine atom does not form any strong interactions (I–I = 4.03 Å, Ni–I = 3.72 Å). The free energy barrier (calculated relative to complex **6-S**) for the RA pathway is 11.0 kcal mol^–1^, which is 6.6 kcal mol^–1^ higher than the EC pathway and 6.0 kcal mol^–1^ higher than the REC pathway. The RA pathway will not compete with the EC and REC pathways, so we will not discuss it in more detail, but we include its full computational data in the ESI (see Fig. S2†).

#### Catalytic cycle

Extensive analysis of these reaction pathways shows that they converge to common Ni(ii)–I intermediates **8-S** on the singlet surface and **8-T** on the triplet surface (Fig. 8). The high spin Ni(ii)–I intermediate **8-T** is lower in free energy than its singlet analogue **8-S** by 14.2 kcal mol^–1^, indicating that the overall C–H iodination reaction has a much larger driving force on the triplet surface than on the singlet surface. From **8-T**, the catalytic cycle is closed by (i) reprotonation of the amide substrate by acetic acid (*i.e.* (AO-*k*^3^-*N*,*N*′,*CI*)Ni(ii)(AcOH)(I) → (AO-*k*^3^-*N*,*NH*′,*CI*)Ni(ii)(OAc)(I)), and (ii) displacement of iodide and the iodinated product (AO–I) by acetates to regenerate the Ni(OAc)_2_ catalyst. If we also invoke the role of the strong base, Na_2_CO_3_, to remove the C–H proton from solution then the overall reaction, Ni(OAc)_2_ (**1-T**) + AO + I_2_ + Na_2_CO_3_ → Ni(OAc)_2_ (**1-T**) + AO–I + NaI + NaHCO_3_, becomes exergonic by 20.8 kcal mol^–1^.

In summary, consideration of several reaction pathways for Ni–C bond iodination with I_2_ reveals that the redox neutral Ni(ii)/Ni(ii) electrophilic cleavage (EC) and Ni(ii)/Ni(iii) single electron reductive electrophilic cleavage (REC) pathways are the most likely mechanisms for this reaction. The computed barrier for the EC pathway is the lowest for the AO substrate, but the computed barrier for the REC pathway is only 0.6 kcal mol^–1^ higher. The previously studied 2-electron Ni(ii)/Ni(iv) oxidative addition/reductive elimination (OA) and Ni(ii)/Ni(iii) radical (RA, homolytic cleavage) pathways are found to be higher in energy for I_2_. Given that the reactivity is highly dependent on the identity of the oxidant/electrophile, these results suggest that the EC and REC pathways should also be considered for Ni-catalyzed C–H functionalization reactions.

### Ni(ii)-catalyzed C–H iodination of 8-aminoquinoline (AQ) substrate with I_2_

To provide further validation and connection to experiments, we also studied the Ni(ii)-catalyzed C–H bond iodination of the AQ substrate with I_2_. We believe that our calculated results are going to be helpful in understanding and rationalizing experimental findings by Chatani^59^ and Koley,^60^ as well as in the prediction of novel ligands. In general, we find that the AQ substrate gives qualitatively the same results as the AO substrate with a few interesting differences that will be discussed here briefly. Full details of the calculations with the AQ substrate can be found in the ESI (see Fig. S3†).

**Fig. 11:**
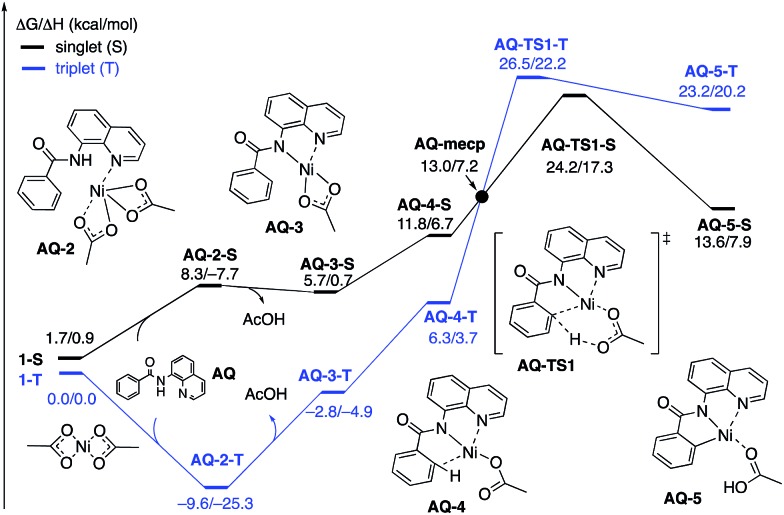
The singlet (black) and triplet (blue) free energy surfaces for C–H bond activation in the AQ substrate by a Ni(OAc)_2_ catalyst. The energies are reported as Δ*G*/Δ*H* in kcal mol^–1^.

**Fig. 12:**
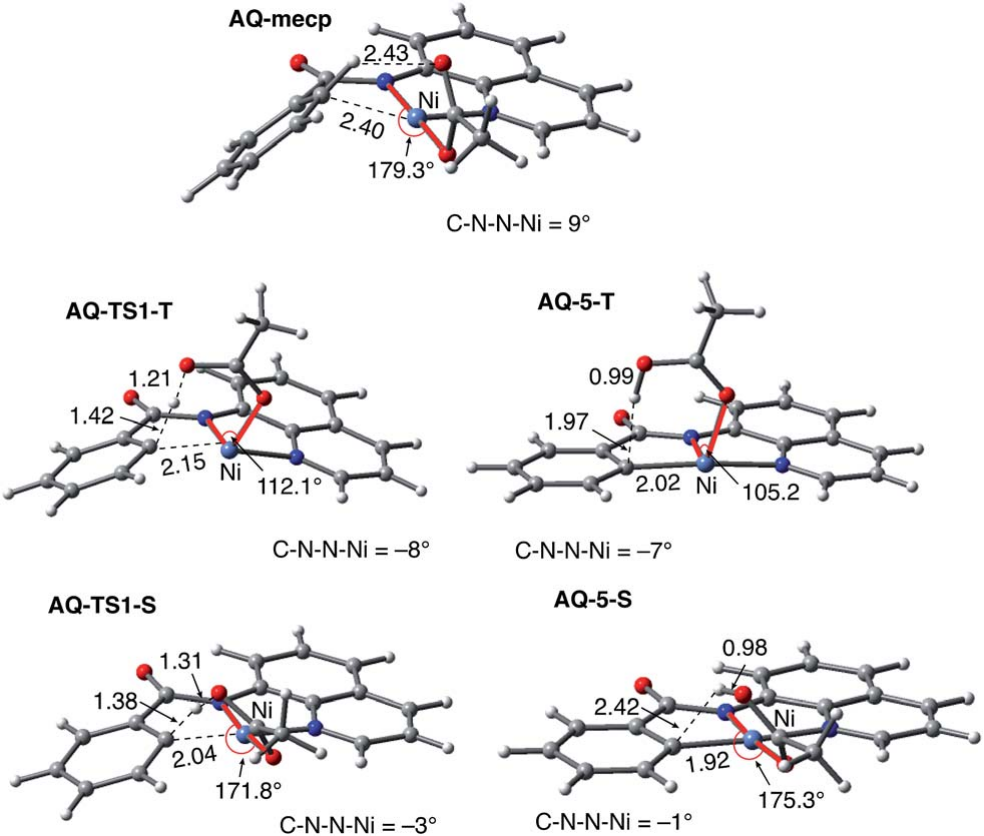
Structural features of the C–H activation transition states with the AQ substrate (**AQ-TS1-T** and **AQ-TS1-S**) and the products (**AQ-5-T** and **AQ-5-S**) on the triplet and singlet surfaces. The distances are shown in Å and angles are shown in degrees.

Firstly, as shown in the calculated potential energy surface in Fig. 11, the C–H activation of the AQ substrate by Ni(OAc)_2_ requires an overall 33.8 kcal mol^–1^ free energy barrier (calculated relative to the triplet complex **AQ-2-T**) at the transition state **AQ-TS1-S**, which is *ca.* 2 kcal mol^–1^ larger than that reported for the AO substrate (Fig. 4). In contrast to the AO substrate, the rate-limiting C–H activation transition state for the AQ substrate, **AQ-TS1-S**, has a singlet ground electronic state; its triplet state counterpart **AQ-TS1-T** lies 2.3 kcal mol^–1^ higher. Thus, the singlet-triplet surface crossing likely occurs before the rate-limiting C–H activation transition state for the AQ substrate. Indeed, we were able to locate a minimum energy crossing point (**AQ-mecp**) that is close in energy (13.0 kcal mol^–1^) and geometry to the singlet reactant structure **AQ-4-S** (Fig. 11 and 12). The nature of the substrate also has a significant impact on the stability of the nickelacycles resulting from the C–H activation. As mentioned above, for the AO substrate, the overall process, Ni(OAc)_2_ (triplet) + AO → **5-S**, is endergonic by 19.5 kcal mol^–1^. In contrast, this process for the AQ substrate, *i.e.* the Ni(OAc)_2_ (triplet) + AQ → **AQ-5-S** reaction, is endergonic only by 13.6 kcal mol^–1^.

Thus, the replacement of the AO substrate by the AQ substrate (a) shifts the C–H bond activation reaction to the singlet surface and the triplet-to-singlet surface crossing occurs before the C–H activation transition state, (b) makes the overall process thermodynamically more favorable by 5.9 kcal mol^–1^ and (c) only slightly (*ca.* 2 kcal mol^–1^) increases the rate-limiting C–H activation barrier. The thermodynamic preference of the C–H activation in AQ compared to AO can be explained by the careful analysis of the geometries in the corresponding final products. Indeed, nickelacycle **AQ-5-S** has a square planar geometry with a ∠(Ni–N–N–C) angle of –1° through the formation of a fused [3.3.0] ring system (Fig. 12), whereas the nickelacycle **5-S** is more twisted out of plane (Ni–N–N–C = –6°) because of additional ring strain introduced by the larger [4.3.0] fused ring system (Fig. 6). This analysis is consistent with the findings of Chen and coworkers.^50^,^51^

Secondly, in contrast to the AO substrate, for the AQ substrate the triplet I_2_-coordination complex **AQ-6-T**, which lies 4.7 kcal mol^–1^ higher than reactants **AQ-1-T** + I_2_, is slightly lower (by 0.5 kcal mol^–1^) than its singlet counterpart **AQ-6-S** (see ESI, Fig. S3†). Likewise, the Ni(ii)/Ni(iii) single electron reductive electrophilic cleavage (REC) free energy barrier (Path-C, initiated from the **AQ-6-T** complex) at the transition state **AQ-TS2-T** is calculated to be lower than the redox neutral Ni(ii)/Ni(ii) electrophilic cleavage (EC) free energy barrier (Path-A, initiated from the **AQ-6-S** complex and following *via* the transition state **AQ-TS2-S**). Based on these results, it appears that the C–H iodination reaction in the AQ substrate will revert back to the triplet surface much earlier on the reaction coordinate (*i.e.* during I_2_ coordination) than that was the case with the AO substrate. However, like the AO substrate, the Ni(ii)/Ni(iii) single electron reductive electrophilic cleavage (REC) and redox neutral Ni(ii)/Ni(ii) electrophilic cleavage (EC) pathways remain close in energy for the AQ substrate. This suggests that one can switch between the substrate structures (or reaction conditions) and still achieve C–I bond formation. Significantly, these data clearly demonstrate the importance of the availability of the lowest-lying electronic states of the first-row transition metal centers for C–H iodination in substrates with an *N*,*N*′-bidentate chelating groups: the actual mechanism of the reaction directly relates to stability and the energy difference between lowest high- and low-spin electronic states.

## Conclusions

**Fig. 13:**
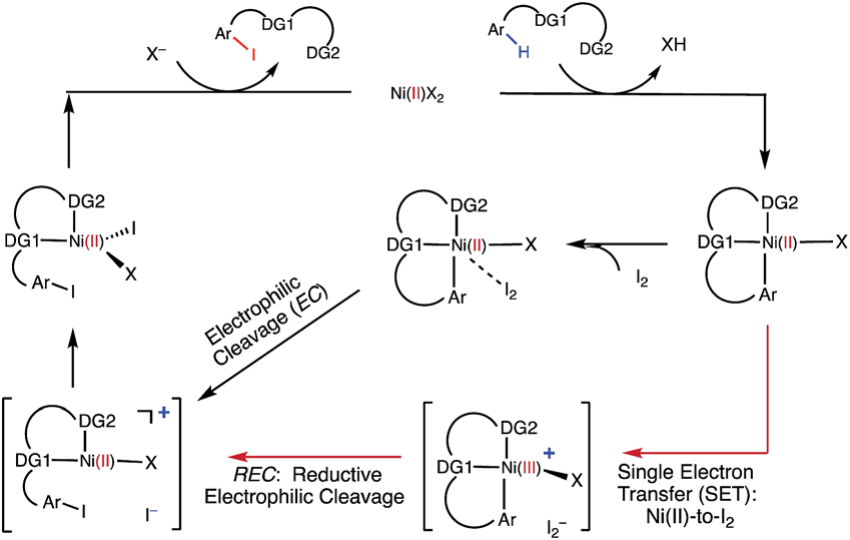
The mechanism proposed for Ni-catalyzed C–H iodination with I_2_ based on the DFT calculations in this study.

Extensive calculations on the elementary steps of Ni(ii)-catalyzed C–H iodination with I_2_ and two substrates with *N*,*N*′-bidentate directing groups (AO and AQ) have revealed the most likely reaction mechanism as illustrated in Fig. 13.

Importantly, we found the relative stability of the lowest energy high- and low-spin electronic states to be an important factor for all of the steps in the reaction. We expect this to be a general feature of first-row transition metal catalysts in C–H functionalization. We found that:

(1) The reaction is initiated by substrate coordination to the triplet Ni(OAc)_2_ complex and N–H deprotonation to form a stable triplet (SUB-*k*^2^-*N*,*N*′,*CH*)Ni(ii)(OAc) complex. The calculated stabilization energies are 6.4 and 9.6 kcal mol^–1^ for the AO and AQ substrates, respectively.

(2) From (SUB-*k*^2^-*N*,*N*′,*CH*)Ni(ii)(OAc), C–H activation occurs *via* the base-assisted CMD mechanism on either the triplet surface (for the AO substrate) or the singlet surface (for the AQ substrate) and generates singlet Ni(ii)-nickelacycles. This process requires a significant free energy barrier (31.8 kcal mol^–1^ for AO and 33.8 kcal mol^–1^ for AQ), occurs *via* triplet-to-singlet spin crossover and is endergonic by 19.5 kcal mol^–1^ for AO and 13.6 kcal mol^–1^ for AQ. Thus, in the absence of an oxidant (or coupling partners) this C–H activation process is not feasible, which is consistent with experiments.^60^

(3) However, in the presence of I_2_ as an oxidant, the coordination of I_2_ to Ni(ii)-nickelacycle provides additional stability to the C–H activation product. In both the singlet and triplet states of the resulting nickelacycle–I_2_ complex **6**, I_2_ accepts electron density from the Ni complex. Since in the triplet state nickelacycle–I_2_ complex **6-T** almost one electron is transferred to I_2_, it was characterized as a [Ni(iii)^+^–I_2_^–^] ion pair complex.

(4) The subsequent C–I bond formation is very fast through either the redox-neural EC pathway, if the reaction starts from the singlet **6-S** complex, or the one-electron REC pathway, if the reaction starts from the triplet **6-T** complex. Both pathways lead to the formation of a stable, high spin Ni(ii)–I intermediate.

(5) The addition of basic Na_2_CO_3_ to the reaction mixture initiates AcO-to-NaCO_3_ ligand exchange and generates the (SUB-*k*^2^-*N*,*N*′,*CH*)Ni(ii)(NaCO_3_) active catalyst. This ligand exchange reaction is exergonic for the AO substrate and requires an insignificant energy barrier. Furthermore, the involvement of a new base, *i.e.* Na_2_CO_3_, reduces the rate-limiting C–H activation barrier by 3.1 kcal mol^–1^ and stabilizes the C–H activation product by 6.3 kcal mol^–1^. These findings are consistent with experiments, showing that Na_2_CO_3_ helps facilitate Ni-catalyzed C–H iodination with I_2_.^59^,^60^

(6) The replacement of the AO substrate by the AQ substrate affects the energy difference between the lowest high- and low-spin electronic states of the systems in several places along the reaction pathway. It makes the C–H activation step thermodynamically more favorable by 5.9 kcal mol^–1^ and only slightly (*ca.* 2 kcal mol^–1^) increases the rate-limiting C–H activation barrier. Thus, the computations indicate that the AO substrate is also viable for Ni(ii)-catalyzed C–H iodination with I_2_.

## Computational details

The calculations were performed with the Gaussian 09 (G09) program.^93^ The geometry optimizations and frequency calculations for all of the reported structures were performed at the B3LYP-D3/[6-31G(d,p) + Lanl2dz (Pd, I)] level of theory with the corresponding Hay–Wadt effective core potential for Pd and I,^94^–^96^ and Grimme’s empirical dispersion-correction (D3) for B3LYP.^97^ Each reported minimum has zero imaginary frequencies and each transition state (TS) structure has only one imaginary frequency. Intrinsic reaction coordinate (IRC) calculations were performed for selected transition state structures to confirm their identity. Bulk solvent effects were incorporated for all of the calculations using the self-consistent reaction field polarizable continuum model (IEF-PCM)^98^–^100^ with dimethylsulfoxide (DMSO) as the solvent. The calculated Gibbs free energies were corrected to a solution standard state of 1 M at room temperature (298.15 K).^101^,^102^

It is known that Ni-complexes may have several energetically close lower-lying electronic states,^63^ therefore, here we investigated both the ground and first excited states of the reactants, intermediates, transition states and products of the reaction. Some of the structures on the singlet potential energy surface had lower energy open-shell singlet electronic states. In these cases, we re-calculated the geometries and energies of the structures at their open-shell singlet electronic states using unrestricted DFT (UB3LYP-D3).^103^,^104^ The minimum energy crossing points (MECP) between singlet and triplet states were located using the MECPro program (v. 1.0.3) developed by Ess and coworkers^105^ with G09.

## Conflicts of interest

There are no conflicts to declare.

## Supplementary Material

Supplementary information

Supplementary information
