# Supplementary material for: The mechanism of directed Ni(ii)-catalyzed C–H iodination with molecular iodine
Source: Chem Sci. 2017 Nov 28;9(5):1144–54. doi: 10.1039/c7sc04604a (PMC5883947; doi:10.1039/c7sc04604a)
Supplement: Supplementary file 1 [file SC-009-C7SC04604A-s001.pdf]

Electronic Supplementary Material (ESI) for Chemical Science.
This journal is © The Royal Society of Chemistry 2017

Supporting Information

The Mechanism of Directed Ni(II)-Catalyzed C–H Iodination with Molecular Iodine

Brandon E. Haines,[a)] Jin-Quan Yu,[b)] Djamaladdin G. Musaev*,[a)]

a) Cherry L. Emerson Center for Scientific Computation, Emory University, 1515 Dickey Drive, Atlanta, Georgia 30322, United States

b) Department of Chemistry, The Scripps Research Institute, 10550 North Torrey Pines Road, La Jolla, California 92037

E-mail: dmusaev@emory.edu

Table of Contents

1. Structures with Base Additive: Na_2CO_3 .... S2
2. Full Energy Surface for Iodination Pathways B and D S3
3. Full Energy Surface for Iodination Pathways A and C with AQ Substrate S5
4. Energies for Calculated Structures S7

1. **Structures with Base Additive: Na_2CO_3**

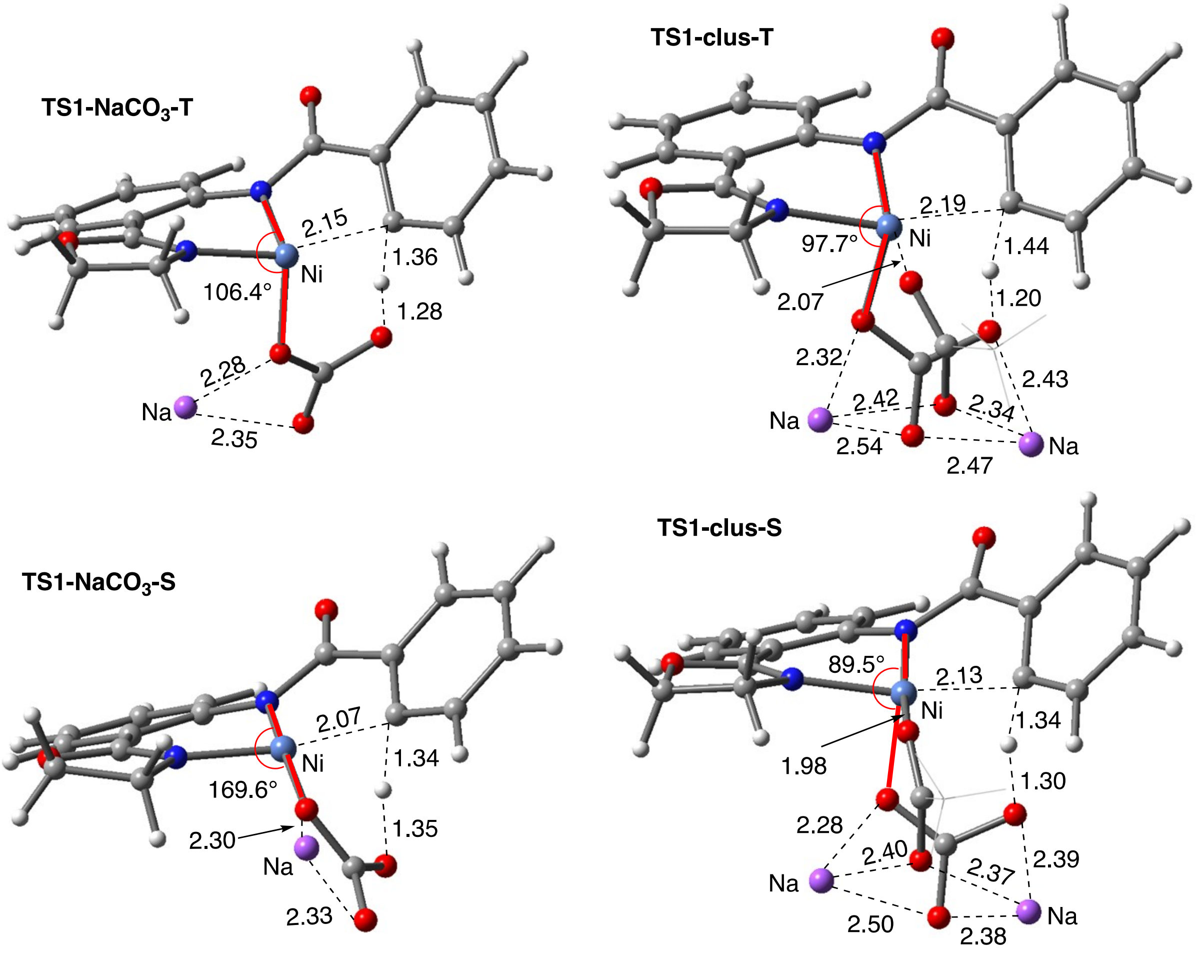

Figure S1. Structures of the C–H activation transition states on the triplet (**TS1-NaCO$_3$-T** and **TS1-clus-T**) and singlet surfaces (**TS1-NaCO$_3$-S** and **TS1-clus-S**) in the presence of base additive, Na_2CO_3. Distances and angles are shown in Å and deg., respectively.

2. Full Energy Surface for Iodination Pathways B and D

The full free energy surfaces computed for the higher energy iodination pathways (Path-B and Path-D) are shown in Figure S2 and are discussed in more detail here.

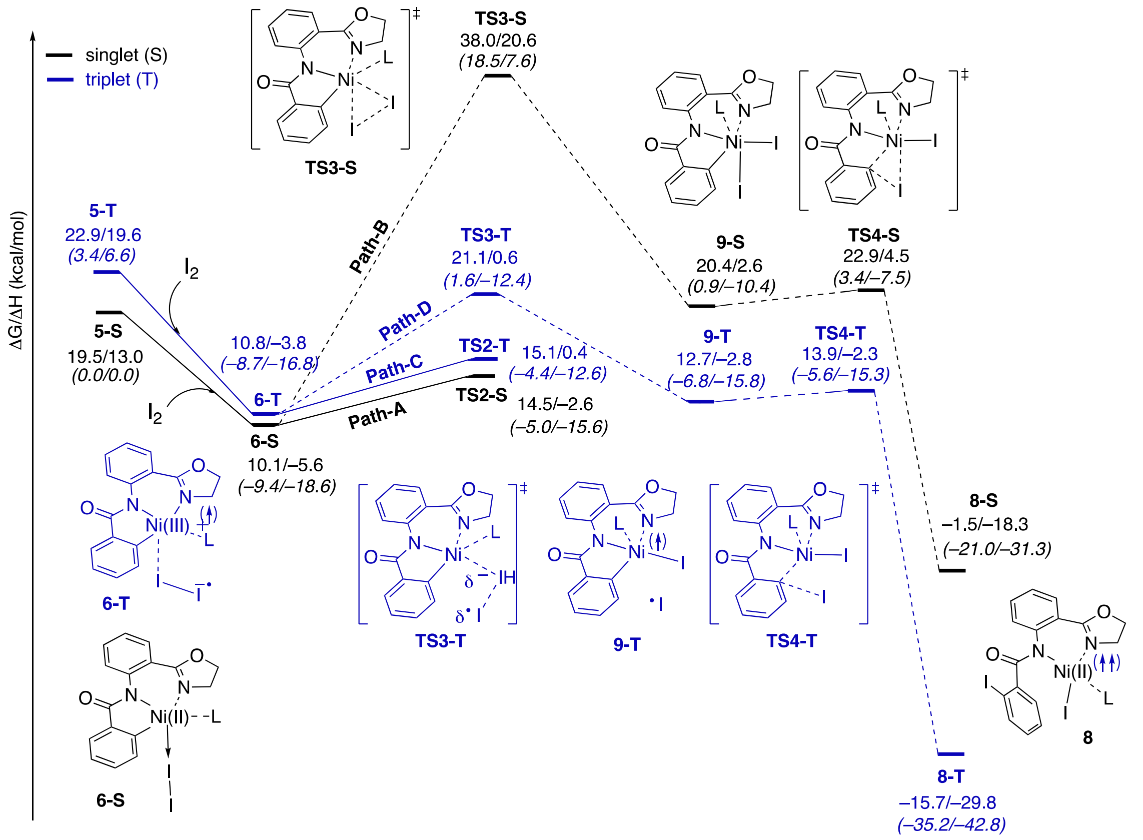

Figure S2. The full singlet (black) and triplet (blue) free energy surfaces for AO substrate C–H bond iodination pathways B and D. Energies are reported as ΔG/ΔH in kcal/mol. Numbers given in first and second lines are relative to dissociation limits of $Ni(OAc)_2$ (triplet) + AO + I_2 and **5-S** + I_2, respectively. Here, L stands for AcOH.

Path-B: Ni(II)/Ni(IV) 2-electron oxidation pathway This pathway starts by oxidative insertion of Ni(II) into the I–I bond at the transition state **TS3-S.** The free energy barrier associated with this oxidative addition transition state is 27.9 kcal/mol, which is 23.5 kcal/mol higher than that required for electrophilic cleavage (EC) pathway (Path-A). The octahedral Ni(IV) product (**9-S**) is endergonic by 10.3 kcal/mol (relative to **6-S**). As expected, the subsequent C–I reductive elimination step is very fast with a free energy barrier of 2.5 kcal/mol (**TS4-S**). Path-B converges to the same product as Path-A (**8-S**).

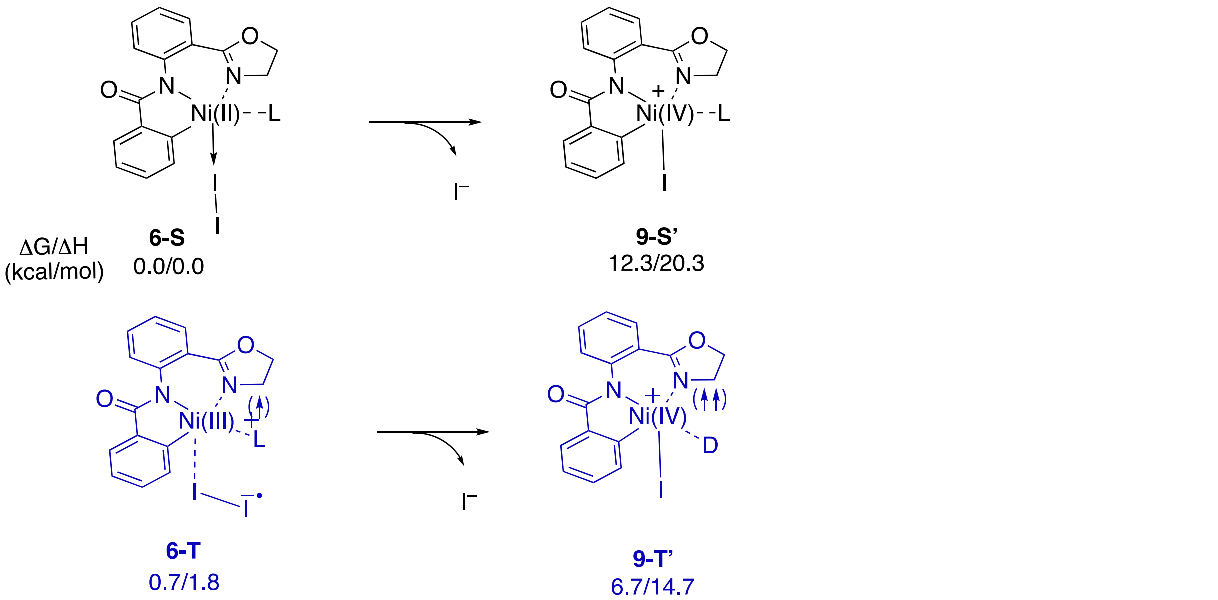

Figure S3. Estimation of oxidation barriers by dissociation of I⁻ from **6-S** and **6-T**. Here, L stands for AcOH.

We also considered dissociation of I⁻ from **6-T** or **6-S** as a mechanism for two electron oxidation, which leads to low and high spin Ni(IV)$^+$ intermediates (**9-S'** and **9-T'**), respectively. We estimate the lower limit of these barriers by calculating the dissociation limit of iodide from these complexes, as shown in Figure S3. We find that the estimated lower bound for the barriers to I⁻ dissociation for each case (ΔG = 12.3 and 6.8 kcal/mol for **6-S** and **6-T**, respectively) are greater than the computed EC and REC barriers ($\Delta G^{\ddagger}$ = 4.4 and 5.0 kcal/mol, respectively).

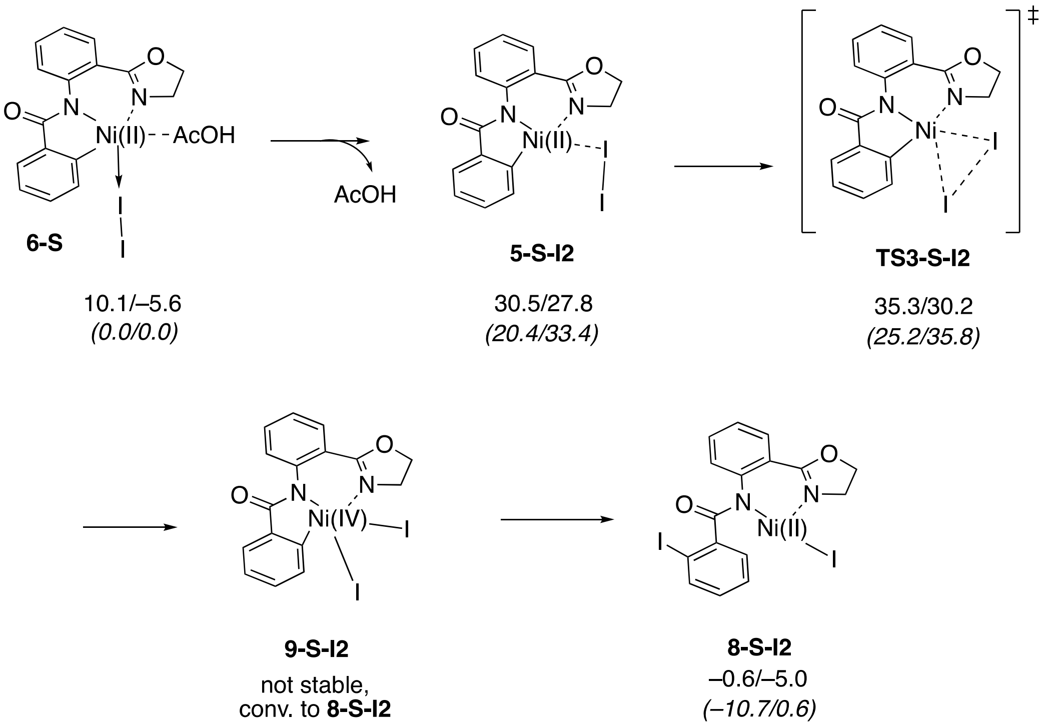

Figure S4. Mechanistic possibility that AcOH dissociates from the nickelacycle prior to oxidative addition of I_2. Energy values given in first and second lines are relative to dissociation limit of $Ni(OAc)_2$ (triplet) + AO + I_2 and **6-S**, respectively.

We also investigated the possibility of dissociation of L, AcOH in this case, from **6-S** to facilitate oxidative addition. Dissociation of the AcOH ligand from **6-S** endergonic by 20.4 kcal/mol. The subsequent oxidative insertion barrier is very low (**5-S-I2** → **TS3-S-I2**, $\Delta G^{\ddagger}$ = 5.8 kcal/mol). The overall barrier computed from **6-S** is 25.2 kcal/mol, which is 2.7 kcal/mol lower than if the AcOH ligand does not dissociate (**6-S** → **TS3-S**, $\Delta G^{\ddagger}$ = 27.9 kcal/mol). This process is still much higher than the EC and REC pathways.

Path-D: Ni(II)/Ni(III) single electron radical pathway (homolytic cleavage) This pathway is also initiated by the one-electron oxidized **6-T** intermediate. In the next step, the I–I bond of I_2^- is cleaved through iodide abstraction by the cationic Ni(III) center (i.e., charge recombination). The free energy barrier (calculated relative to complex **6-S**) for the RA pathway is 11.0 kcal/mol, which is 6.6 kcal/mol higher than the EC pathway and 6.0 kcal/mol higher than the REC pathway. The product is radical pair between Ni(III)–I and iodine atom (**9-T**) that is endergonic by 2.6 kcal/mol (relative to **6-S**). Subsequent cleavage of the Ni(III)–C bond by the iodine radical is also very fast with a free energy barrier of 1.2 kcal/mol (**TS4-T**). Path-D converges to the same product as Path-C (**8-T**).

3. Full Energy Surface for Iodination Pathways A and C with AQ Substrate

The full free energy surfaces computed for the iodination pathways with substrate AQ (Path-A and Path-C) are shown in Figure S3 and are discussed in more detail here.

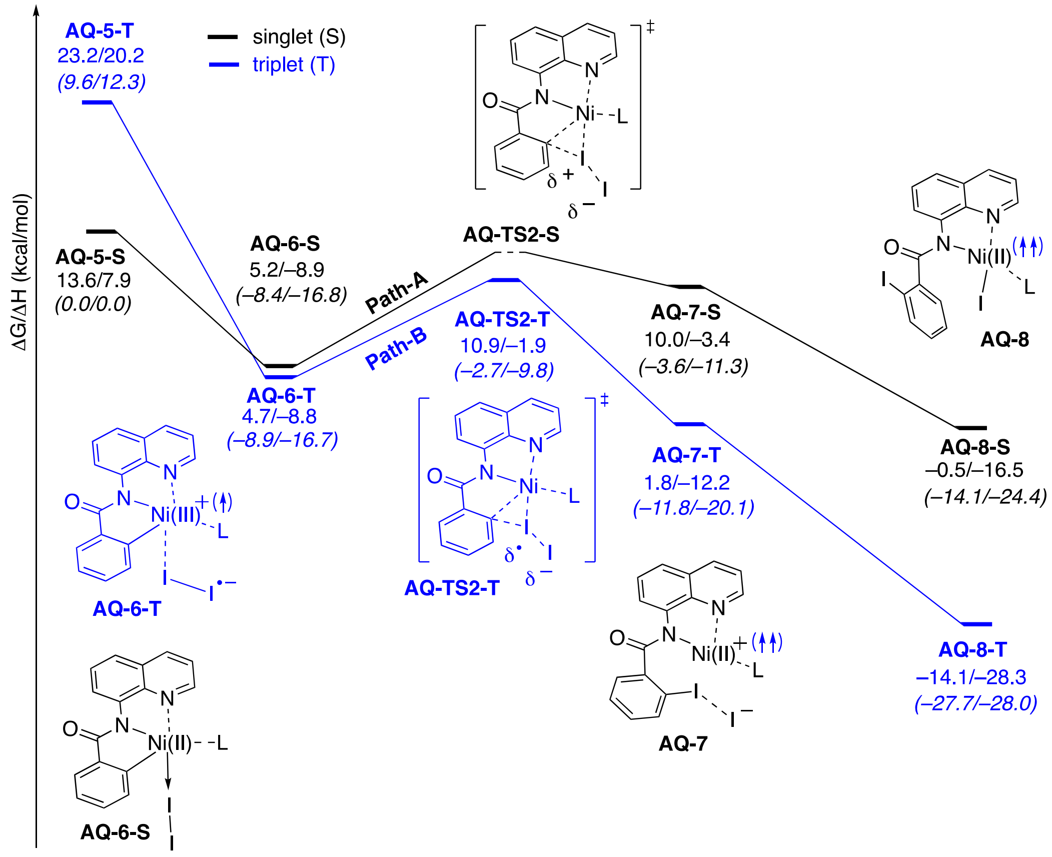

Figure S5. The singlet (black) and triplet (blue) free energy surfaces for AQ substrate C–H bond iodination pathways A and B. Energies are reported as $\Delta G/\Delta H$ in kcal/mol. Numbers given in first and second lines are relative to dissociation limits of $Ni(OAc)_2$ (triplet) + AQ + I_2 and **AQ-5-S** + I_2, respectively. Here, L stands for AcOH.

Path-A: Redox neutral Ni(II)/Ni(II) electrophilic cleavage (EC) mechanism. The TS for the EC pathway with substrate AQ (**AQ-TS2-S**) could not be located. However, the energy of its product complex (**AQ-7-S**, 5.3 kcal/mol relative to **AQ-6-T**) can serve as the lower bound of the free energy barrier. The estimated free energy barrier is higher but comparable to that computed for the AO substrate (4.4 kcal/mol). Subsequent combination of the $Ni(II)^+$ and iodide ions to produce the Ni(II)–I intermediate **AQ-8-S** is exergonic (by 5.7 kcal/mol).

Path-C: Ni(II)/Ni(III) single electron reductive electrophilic cleavage (REC). Again, the one-electron oxidation process of converting **AQ-6-S** to **AQ-6-T** initiates this pathway, except with AQ, the triplet state structure is lower in energy (by 0.5 kcal/mol). The free energy barrier (calculated relative to complex **AQ-6-T**) for the REC pathway is found to be 6.2 kcal/mol, which is 1.2 kcal/mol higher than the REC pathway for the AO substrate. Formation of the product complex, **AQ-7-T**, is exergonic by 2.9 kcal/mol, and combination of the $Ni(II)^+$ and iodide ions to produce **AQ-8-T** is exergonic by 18.8 kcal/mol.

4. **Energies for Calculated Structures**

The electronic energies (in hartrees) calculated at B3LYP-D3/BS1 [6-31G(d,p) + Lanl2dz (Pd, I)] level of theory are provided, as well as the applied zero point energy (ZPE), enthalpy (H), and Gibbs free energy (G°) corrections calculated at the B3LYP-D3/BS1 level for all structures. The calculated Gibbs free energies are corrected to a solution standard state of 1M at 298.15 K. Bulk solvent effects are incorporated for all calculations using the self-consistent reaction field polarizable continuum model (IEF-PCM) with dimethylsulfoxide as the solvent. Imaginary frequencies are provided for transition state structures.

Table S1. Energies for C–H Iodination reaction pathways with AO substrate

Structure	E(BS1)	ZPE	H	G°	$\langle S^2 \rangle$	Im. Freq.
**1-S**	–626.362947	0.103450	0.115083	0.065056	-	none
**1-T**	–626.363752	0.102265	0.114527	0.063186	2.004	none
**AO**	–878.170514	0.271683	0.288892	0.228172	-	none
**AcOH**	–229.101006	0.061765	0.067255	0.037709	-	none
**2-S**	–1504.550324	0.377901	0.406966	0.319936	-	none
**2-T**	–1504.573135	0.378056	0.407527	0.316986	2.004	none
**3-S**	–1275.430516	0.313468	0.336765	0.262792	-	none
**3-T**	–1275.439166	0.312662	0.336293	0.260750	2.005	none
**4-S**	–1275.425986	0.314370	0.337497	0.264381	-	none
**4-T**	–1275.424262	0.312145	0.336242	0.258178	2.006	none
**TS1-S**	–1275.395270	0.309135	0.331566	0.260797	-	1416.7i
**TS1-T**	–1275.394822	0.307299	0.330485	0.255768	2.006	1200.5i
**5-S**	–1275.413786	0.314491	0.337405	0.265298	-	none
**5-S-I2**	–1069.059667	0.251212	0.273391	0.197999	-	none
**5-T**	–1275.401490	0.311802	0.335653	0.258433	2.007	none
**I_2**	–22.771490	0.000410	0.004307	–0.022538	-	none
**6-S**	–1298.216720	0.315773	0.343563	0.256184	-	none
**6-T**	–1298.213731	0.315397	0.343362	0.254272	2.049	none
**TS2-S**	–1298.211265	0.315751	0.342844	0.257643	-	44.3i
**TS2-T**	–1298.205317	0.313975	0.341721	0.252726	2.187	134.5i
**7-S**	–1298.221841	0.317341	0.344988	0.256526	-	none
**7-T**	–1298.224424	0.315190	0.343552	0.252554	2.005	none
**7-S-I3**	–1321.037601	0.318905	0.350995	0.250445	-	none
**7-T-I3**	–1321.033160	0.316317	0.349528	0.243553	2.004	none
**8-S**	–1298.236989	0.315957	0.343587	0.257933	-	none
**8-S-I2**	–1069.112647	0.252207	0.274107	0.201343	-	none
**8-T**	–1298.254267	0.314112	0.342486	0.252639	2.006	none
**AO–I**	–888.925145	0.260845	0.279781	0.213186	-	none
**NaI**	–173.822087	0.000209	0.004264	–0.022167	-	none
**TS3-S**	–1298.173538	0.314906	0.342027	0.257425	-	82.8i
**TS3-S-I2**	–1069.054939	0.251154	0.272508	0.200815	-	37.5i
**TS3-T**	–1297.891041	0.314317	0.341573	0.255743	2.16	28.9i
**9-S**	–1298.202960	0.315330	0.342766	0.258830	-	none
**9-T**	–1297.896646	0.314657	0.342630	0.254911	2.12	none
**I^-**	–11.571872	0.000000	0.002360	–0.013830	-	none
**I_3^-**	–34.377465	0.000621	0.006661	–0.024909	-	none
**9-S’**	–1286.612146	0.315512	0.340860	0.260783	-	none
**9-T’**	–1286.620558	0.314685	0.340373	0.259399	2.066	none
**TS4-S**	–1298.197024	0.314236	0.341530	0.256908	-	130.6i
**TS4-T**	–1297.895751	0.312211	0.340008	0.253506	2.27	442.7i

Table S2. Energies for C–H activation in presence of Na_2CO_3 additive

Structure	E(BS1)	ZPE	H	G°	<S²>	Im. Freq.
**Na_2CO_3**	–588.580374	0.016597	0.024316	–0.016103	-	none
**$NaHCO_3$**	–426.812773	0.027639	0.034005	–0.001865	-	none
**NaOAc**	–390.885920	0.049857	0.056217	0.020496	-	none
**3-clus-S**	–1864.073623	0.330745	0.362488	0.266484	0.630	none
**3-clus-T**	–1864.088982	0.330104	0.362020	0.264392	2.004	none
**TS1-clus-S**	–1864.036568	0.325754	0.356825	0.263473	0.680	1306.9i
**TS1-clus-T**	–1864.050367	0.325375	0.356622	0.261311	2.005	1028.7i
**5-clus-S**	–1864.076038	0.332191	0.363357	0.270371	-	none
**5-clus-T**	–1864.058112	0.329875	0.361843	0.263238	2.005	none
**3-$NaCO_3$-S**	–1473.157171	0.279801	0.303680	0.225714	-	none
**3-$NaCO_3$-T**	–1473.154601	0.278859	0.302388	0.223649	2.005	none
**TS1-$NaCO_3$-S**	–1473.110106	0.274890	0.298214	0.222291	0.158	1277.4i
**TS1-$NaCO_3$-T**	–1473.112219	0.273093	0.297116	0.215648	2.005	1301.7i
**5-$NaCO_3$-S**	–1473.136642	0.279571	0.303600	0.225228	-	none
**5-$NaCO_3$-T**	–1473.120424	0.277582	0.302268	0.219999	2.007	none

Table S3. Energies for C–H Iodination reaction pathways with AQ substrate

Structure	E(BS1)	ZPE	H	G°	<S²>	Im. Freq.
**AQ**	–801.749363	0.244500	0.259686	0.204685	-	none
**AQ-2-S**	–1428.128488	0.350043	0.377385	0.293486	-	none
**AQ-2-T**	–1428.156253	0.349572	0.377077	0.292697	2.004	none
**AQ-3-S**	–1199.011766	0.286422	0.307653	0.238842	-	none
**AQ-3-T**	–1199.019917	0.285062	0.307018	0.233555	2.004	none
**AQ-4-S**	–1199.002078	0.286254	0.307535	0.238964	-	none
**AQ-4-T**	–1199.006175	0.284903	0.306978	0.234249	2.005	none
**AQ-mecp (singlet)**	–1198.999851	0.285386	0.306207	0.238630	-	21.1i
**AQ-TS1-S**	–1198.979824	0.281685	0.302171	0.236476	-	1366.4i
**AQ-TS1-T**	–1198.970957	0.279834	0.301133	0.231285	2.005	1215.6i
**AQ-5-S**	–1199.000603	0.286866	0.307988	0.240321	-	none
**AQ-5-T**	–1198.979399	0.284516	0.306447	0.234350	2.007	none
**AQ-6-S**	–1221.800531	0.287987	0.314062	0.229873	-	none
**AQ-6-T**	–1221.800332	0.287777	0.313920	0.228896	2.038	none
**AQ-TS2-T**	–1221.787341	0.285817	0.311940	0.225768	2.178	146.2i
**AQ-7-S**	–1221.792470	0.288592	0.314733	0.229461	-	none
**AQ-7-T**	–1221.806302	0.288442	0.314612	0.230141	2.005	none
**AQ-8-S**	–1221.812831	0.288623	0.314245	0.232993	-	none
**AQ-8-T**	–1221.831777	0.288194	0.314279	0.230272	2.005	none
